# Draft Genome Sequence of *Solibacillus kalamii*, Isolated from an Air Filter Aboard the International Space Station

**DOI:** 10.1128/genomeA.00696-17

**Published:** 2017-08-31

**Authors:** Arman Seuylemezian, Nitin K. Singh, Parag Vaishampayan, Kasthuri Venkateswaran

**Affiliations:** Biotechnology and Planetary Protection Group, Jet Propulsion Laboratory, California Institute of Technology, Pasadena, California, USA

## Abstract

We report here the draft genome of *Solibacillus kalamii* ISSFR-015, isolated from a high-energy particulate arrestance filter aboard the International Space Station. The draft genome sequence of this strain contains 3,809,180 bp with an estimated G+C content of 38.61%.

## GENOME ANNOUNCEMENT

*Solibacillus* is a novel microbial genus characterized in 2009 by Krishnamurthi et al., who reclassified *Bacillus silvestris* into the genus *Solibacillus*, making it the type species (1). The *Solibacillus* genus was described as comprising *Bacillus*-like organisms with Gram-positive, rod-shaped cells, and round endospores formed terminally in swollen sporangia. In 2016, Poonam et al. reclassified *Bacillus isronensis* into the genus *Solibacillus* and provided an emended description of the genus *Solibacillus* that showed the peptidoglycan type to be A4α-l Lys-d-Glu and the fatty acid profile to contain iso-C_15:0_, C_16:1_ω7c alcohol, and iso-C_17:1_ω7c, with MK-7 being the major menaquinone and diphosphatidylglycerol, phosphatidylglycerol, phosphatidylethanolamine, and phosphatidylserine being the major polar lipids (2).

Until recently, there were two bacterial species in the genus *Solibacillus* (*S. isronensis* and *S. silvestris*), and draft genomes of both species have been completed (3, 4). In an ongoing microbiological survey from 2014 to 2017, a novel bacterial species was isolated from a high-energy particulate arrestance (HEPA) filter system aboard the International Space Station (ISS). The round spore-forming bacterium was described as *Solibacillus kalamii* (5), and here we present its draft genome. The HEPA filter was in service for 40 months on the ISS before being brought back to earth for further microbiological and molecular analyses (5).

In this study, we characterize the draft whole-genome sequence of strain *S. kalamii* ISSFR-015 (NRRL 65388^T^ = DSM 101595^T^), which provides insight on putative genes coding for superoxide dismutase, universal stress proteins, vancomycin resistance, copper resistance, hydrogen peroxide resistance, and arsenic resistance. This evidence is suggestive of the bacterium’s success in surviving the high-stress environment of the ISS and the harsh cleaning procedures during spacecraft assembly.

*S. kalamii* strain ISSFR-015^T^ was sequenced following a shotgun sequencing method using the Illumina MiSeq platform with a paired-end module. A total of 12,244,464 paired-end reads were generated, and CLC Genomics Workbench version 10.0.1 was used to filter for adapter-free, high-quality reads, yielding 8,435,258 reads representing an approximate genome coverage of 332×. These reads were assembled using CLC Genomics Workbench, resulting in 28 contigs with a total size of 3,809,180 and an *N*_50_ contig length of 199,024 bp. The largest assembled contig measured 778,658 bp. The NCBI Prokaryotic Genome Annotation Pipeline was used for annotating protein-coding genes and other functional elements present in the genome. The genome contained a total of 3,814 genes, of which 3,662 were protein-coding genes. The complete genome of strain ISSFR-015^T^ was 3,809,180 bp in length with an estimated G+C content of 38.61% and with 64 tRNA genes and 18 rRNA (eight 5S, six 16S, and four 23S) genes.

### Accession number(s).

The genome sequences of *S. kalamii* ISSFR-015 have been deposited at DDBJ/ENA/GenBank under the accession number NHNT00000000.
